# Not as simple as it seems: extensive facility and training gaps in nursing home bathing

**DOI:** 10.1017/ice.2023.109

**Published:** 2023-09

**Authors:** Kristine P. Nguyen, Raveena D. Singh, Raheeb Saavedra, John T. Billimek, Steven P. Tam, Karl E. Steinberg, Lori Porter, John Mitchell, Susan S. Huang

**Affiliations:** 1 Division of Infectious Diseases, University of California, Irvine School of Medicine, Irvine, California; 2 Department of Family Medicine, University of California, Irvine School of Medicine, Irvine, California; 3 Division of Geriatrics and Gerontology, University of California, Irvine School of Medicine, Irvine, California; 4 Shiley Haynes Institute for Palliative Care, California State University–San Marcos, San Marcos, California; 5 National Association of Health Care Assistants, Carl Junction, Missouri

## Abstract

Standardized observation of bed baths and showers for 100 residents in 8 nursing homes revealed inadequate cleansing of body sites (88%–100% failure) and >90% process failure involving lather, firm massage, changing dirty wipes or cloths, and following clean-to-dirty sequence. Insufficient water warmth affected 86% of bathing opportunities. Bathing training and adequate resources are needed.

Each year, ∼3 million healthcare-associated infections occur in US nursing homes (NHs).^1^ NH residents are at high risk for infection due to age, comorbidities, wounds, and medical devices.^2–5^

Many residents have limited ability for self-care and depend on caregivers for hygiene needs including bathing and showering. However, staff bathing training is brief and limited, likely because bathing is assumed to be gained from personal experience. Nevertheless, personal experience means that staff are most comfortable with cleansing intact skin, and possibly avoidant of areas with poor skin integrity, wounds, or devices. We assessed bathing quality and barriers to proper bathing in NHs.

## Methods

We conducted a prospective observational study of bathing in 8 NHs in Orange County, California from September to November 2022, involving a convenience sample of bed baths and showers conducted for quality improvement (QI). Study staff used a standardized observation form to evaluate quality of NH bathing and showering (Supplementary Material online). Survey elements included cleansing of specific body sites (ie, hair, face and neck, skin folds, male or female genitals, fingers and toes) and adherence to bathing and showering procedures (eg, sufficient lather, clean-to-dirty sequence, wrap and unwrap wounds and devices, fully towel dry). The survey also included queries to staff to further assess knowledge and understand perceived barriers (eg, “What do you know now about bathing that you wish you had known when you first started?”). NH staff were told that observation was occurring, and no feedback was given during or after bathing. This study was conducted under QI operations at each NH with assent from cognitively aware residents and was exempt from human-subject review.

Observed lapses were documented along with observer-determined reasons for nonadherence. These reasons included training issues (eg, poor lather, skipped body areas, dirty-to-clean sequence), facility issues (eg, insufficient hot water, inflexible showerhead), a combination of training and facility issues, timing issues (eg, staff called to other tasks, staff expressed need to bathe quickly), and resident issues (eg, refused, combative). Percentage of nonadherence with each element was tabulated for bed baths and showers separately.

## Results

**Figure 1.**
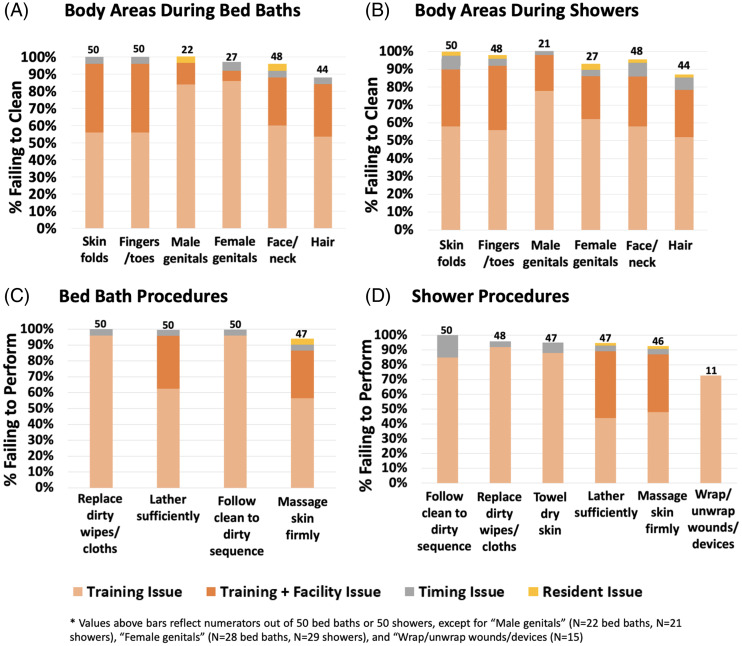
Bar graphs illustrating the frequency (panels A and B) and reasons for failures (panels C and D) during bed baths and showers. Panels A and B display the percentage of bed baths and showers where failure to clean specific body areas was observed. All NHs used regular soap and water. Panels C and D display the percentage of bed baths and showers where failures to adhere to standard procedures was observed. Failures were most commonly attributed to training, facility, or training and facility issues.

In total, 50 bed baths (NH range, 5–8) and 50 showers (NH range, 4–7) were observed across 8 NHs. Lapses in bathing quality and process were extremely common (Fig. 1). Inadequate body cleansing occurred for all observed body sites, ranging from 88% to 100% failure to clean for both bed baths and showers. Notably, failure to clean male or female genitalia was >90%. Most body areas were skipped or only sprayed with water without soaping. Additionally, procedural failures were common for bed baths and showers: insufficient lather [50 (100%) bed bath and 20 (40%) shower], lack of firm massage for cleaning [47 (94%) bed bath and 45 (90%) shower], failure to change soiled wipes or cloths [50 (100%) bed bath and 48 (96%) shower], failure to follow clean-to-dirty sequence [50 (100%) bed bath and 48 (96%) shower]. In addition, frequent failures to wrap and unwrap devices (n = 37, 74%) for showering and failure to fully towel dry (n = 47, 94%) after showering were observed.

Reasons for failure were most often perceived as training issues, facility issues, or both. Observable timing constraints and resident combativeness or refusal were rare. The dominant facility-based issue was related to water temperature, with nearly all residents complaining about being cold (N = 86, 86%). When queried about the most important staff-to-staff bathing advice, staff most mentioned competing for the “better shower” and “bathing earlier in the day to get hot water.”

To evaluate whether complaints of being cold affected the quality of bathing, we stratified the likelihood of inadequate body site cleansing and inaccurate bathing procedures by whether the resident was cold (Table 1). Failing to clean specific body sites was not significantly different between the groups, with the exception that staff were less likely to wash the hair of residents who were cold (89.5% vs 64.3% not washed; *P* = .02). However, sufficiently warm water significantly improved adherence to bathing procedures (46.5% mean failure rate across procedural elements when water was warm versus 79.9% when water was cold). Nevertheless, errors in bathing procedures remained common even with adequate bathing temperatures.

**Table 1.** Body Site and Procedural Failures for Bed Baths and Showers by Whether Residents Complained of Being Cold

Variable	Failures for Bed Baths and Showers^a^	
	Resident Complained of Being Cold, No. % (95% CI)	Resident Did Not Complain of Being Cold, No. % (95% CI)
No. of observed residents	86	14
Duration of bed baths and showers, average minutes^b^	12	14
**Failure to clean body sites**		
Hair	**77** **89.5% (81.1%–95.1%)**	**9** **64.3% (35.1%–87.2%)**
Face/neck	69 80.2% (70.3%–88.0%)	8 57.1% (28.9%–82.3%)
Fingers/toes	78 90.7% (82.5%–95.9%)	11 78.6% (49.2%–95.3%)
Skin folds	64 74.4% (63.9%–83.2%)	9 64.3% (35.1%–87.2%)
Male genitals^c^	34 79.1% (75.2%–97.1%)	3 75.0% (19.4%–99.4%)
Female genitals^d^	37 80.4% (69.9%–93.4%)	7 77.8% (40.0%–97.2%)
% Failures across body sites	82.4	69.5
**Failure to follow procedures for bed baths and showers**		
Replace dirty wipes/cloths	**69** **80.2% (70.3%–88.0%)**	**6** **42.9% (17.7%–71.1%)**
Lather sufficiently	**63** **73.3% (62.6%–82.2%)**	**6** **42.9% (17.7%–71.1%)**
Follow clean to dirty sequence	**76** **88.3% (79.7%–94.3%)**	**8** **57.1% (28.9%–82.3%)**
Massage skin firmly	**67** **77.9% (67.7%–86.1%)**	**6** **42.9% (17.7%–71.1%)**
% Failures across bed bath and shower procedures	79.9	46.5
**Failures in shower-only procedures**		
Fully towel-dried skin^e^	**37** **82.2% (68.0%–92.0%)**	**2** **40.0% (5.3%–85.3%)**
Wrap/unwrap devices^f^	5 83.3% (35.9%–99.6%)	1 100.0% (2.5%–100%)

a Bold indicates *P* < .05 using the Fisher exact test. No correction for multiple comparisons.

b Start time: initial water contact; stop time: initial towel contact.

c Based on denominator of males: N=38 (cold); N=4 (not cold).

d Based on denominator of females: N=44 (cold); N=9 (not cold).

e Based on denominator of those showered: N=45 (cold); N=5 (not cold). Residents were often partially dried (hair, shoulders, legs) with a covering towel prior to returning to their bedroom where full drying was supposed to occur. If they were not toweled off at that time, this was considered a failure.

f Based on denominator of those with devices requiring waterproofing: N=6 (cold); N=1 (not cold).

## Discussion

We identified extensive deficiencies in body cleansing, procedural steps, and water temperature for bed bathing and showering in all NHs surveyed. Suboptimal bathing was the norm. As an integral component of care, bathing is important for hygiene, comfort, confidence in appearance, ensuring healthy skin, and preventing infection. In addition, because NHs are high-risk environments for multidrug-resistant organisms (MDROs),^6,7^ ensuring proper bathing and showering can protect residents from acquiring unwanted pathogens and reduce surface pathogens that cause disease.

Observed reasons for poor-quality bathing were multimodal, driven by both training and facility needs. Training could improve the sufficiency of applied soap. Common errors involved failure to use soap, failure to apply soap to all areas of the body, or failure to massage soap well to remove sweat, grime, and germs. Additional training-based errors included failure to bathe body parts sequentially from clean to dirty, failure to change soiled cloths, and failure to fully towel dry after showering. Bathing was only minimally improved for easily accessible areas such as the face, neck, and male genitalia compared to less accessible areas such as female genitalia, body folds, or between fingers and toes. Similarly, warm water temperature did not appreciably improve cleansing of specific body sites. Altogether, this suggests a fundamental deficiency in bathing training that must be addressed.

Facility issues were dominated by suboptimal water temperature impacting both bed baths and showers, with 9 of 10 residents complaining about being cold. Being cold markedly worsened attention to proper bathing processes and procedures, likely forcing staff to bathe quickly and causing issues with insufficient or nonexistent lather, rapid and incomplete wiping without adequate massage, and use of a single cloth for the whole bath or shower regardless of soilage or clean-to-dirty sequence. Nevertheless, there was still a 45% failure in these procedures when water temperature was adequate, suggesting a need for both improved facilities (sufficient warm/hot water to last the duration of bathing time for the entire facility) and training and education for staff performing bathing. Routine bathing and showering observations to provide feedback to NH leadership about processes and problems are likely needed for corrective response.

This study had several limitations. The study was observational and was conducted in a limited number of NHs. As an observational survey, we did not inquire about training opportunities, supply chain (eg, cloth shortages), or instructions to staff to conserve linens or reduce laundry, which, for example, could explain failure to change soiled cloths. We also conducted the study in a single region, which limits generalizability. Nevertheless, NHs varied in size and proportion of short/long-stay residents and were variably affiliated with corporations.

Overall, extensive gaps in bathing and showering existed across all NHs despite the high risk and complex care setting. High-quality bathing and showering are essential because of high MDRO prevalence and high risk of pathogen transmission in this congregate setting^6–9^ and because residents often have medical devices, wounds (eg, pressure ulcers, surgical wounds), rashes and other causes of nonintact skin.^6,7^ Thus, bathing of NH residents cannot be assumed to be intuitive.^10^ It requires training, feedback, and adequate resources, particularly sufficient hot water. Dedicated and comprehensive educational efforts are needed to ensure that NH staff become skilled in bathing procedures as a critical component of high-quality NH care.

## Supplementary material

For supplementary material accompanying this paper visit https://doi.org/10.1017/ice.2023.109. click here to view supplementary material
