# Supplementary material for: Not as simple as it seems: extensive facility and training gaps in nursing home bathing
Source: Infect Control Hosp Epidemiol. 2023 Jun 16;44(9):1490–3. doi: 10.1017/ice.2023.109 (PMC10507494; doi:10.1017/ice.2023.109)
Supplement: Supplementary file 1 [file S0899823X23001095sup.zip › S0899823X23001095sup001.pdf]

SHOWER Skills & Barrier Assessment

Assessment

Date: ___/___/_____ Staff Initials _____ Resident Room #_____ *Shower START time:* ________*AM/PM* *STOP time:* ______ *AM/PM*

Observed Cleansing Practices

Note: For each observation, if "N" provide observed reason for failure:

1 – Timing issue (rushed, too many demands, etc.)
2 – Resident issue (refused, combative, etc.)
3 – Training issue (unaware of problem, uncertain how to approach)
4 – Other (please describe)

If "No", provide # and likely explanation for failure:

- ☐ Y ☐ N ☐ N/A Devices/wounds waterproofed? ____________________
- ☐ Y ☐ N Sufficient lather created? ____________________
- ☐ Y ☐ N Massaged skin *firmly* ____________________
- ☐ Y ☐ N Cleaned hair ____________________
- ☐ Y ☐ N Cleaned face/neck well, avoided eyes/ears ____________________
- ☐ Y ☐ N Cleaned between all skin folds ____________________
- ☐ Y ☐ N Cleaned between fingers/toes ____________________
- ☐ Y ☐ N ☐ N/A Cleaned open wounds ____________________
- ☐ Y ☐ N ☐ N/A Cleaned over dressings that were not wrapped ____________________
- ☐ Y ☐ N ☐ Cleaned ☐ female or ☐ male genital area well ____________________
- ☐ Y ☐ N Are all parts of the skin towel-dried after the shower? ____________________
- ☐ Y ☐ N Wipes/cloths replaced with new ones when soiled ____________________
- ☐ Y ☐ N Disposed of used wipes in trash/did not flush ____________________
- ☐ Y ☐ N Resident complained about being cold during the shower ____________________

Wrapped Devices & Wrapped Wounds

- ☐ Y ☐ N ☐ N/A After shower, removed waterproofing?____________________
- ☐ Y ☐ N ☐ N/A Notified treatment nurse for cleaning ____________________
- ☐ Y ☐ N ☐ N/A Cleaned occlusive/semi-permeable dressings ____________________
- ☐ Y ☐ N ☐ N/A Unwrapped devices/cleansed ____________________
- ☐ Y ☐ N ☐ N/A Cleaned 6 inches of all tubes/central lines/drains closest to body ____________________
- ☐ Y ☐ N ☐ N/A Cleaned superficial wounds/rash/stage 1 & 2 decubitus ulcers ____________________
- ☐ Y ☐ N ☐ N/A Cleaned surgical wounds (unless primary dressing or packed) ____________________

Comments on Showering Processes

____

____

Query to Bathing Assistant

1. **If your resident has a wrapped device or wound, how would you ensure that it gets cleaned during a shower?**

   Lines/Drains/Tubes ____

   Wounds ____

   Dressings ____

2. **How many cloths do you usually bring for a shower? What type?** ____

3. **What part of your bathing style or skills are you most proud of and why?**

   ____

   ____

4. **What bathing advice would you share with a new staff member that you wish you had known earlier? How did you learn this?**

   ____

   ____

5. **How many years have you worked as a CNA in a nursing home?** ____
