# Supplementary material for: Not as simple as it seems: extensive facility and training gaps in nursing home bathing
Source: Infect Control Hosp Epidemiol. 2023 Jun 16;44(9):1490–3. doi: 10.1017/ice.2023.109 (PMC10507494; doi:10.1017/ice.2023.109)
Supplement: Supplementary file 1 [file S0899823X23001095sup.zip › S0899823X23001095sup002.pdf]

BED BATH Skills & Barrier Assessment

Assessment

Date: ___/___/_____ Staff Initials _____ Resident Room #_____ *Bath START time:* ________*AM/PM* *Bath STOP time:* ________ *AM/PM*

Type of Applicator: ☐ Disposable wipes ☐ Cotton cloths ☐ Other ______________________________

Observed Bathing Practices

Note: For each observation, if "N" provide observed reason for failure:

1 – Timing issue (rushed, too many demands, etc.)
2 – Resident issue (refused, combative, etc.)
3 – Training issue (unaware of problem, uncertain how to approach)
4 – Other (please describe)

If "No", provide # and likely explanation for failure:

☐ Y ☐ N | Sufficient lather created ______________________________

☐ Y ☐ N | Massaged skin *firmly* ______________________________

☐ Y ☐ N | Cleaned hair ______________________________

☐ Y ☐ N | Cleaned face/neck well, avoided eyes/ears ______________________________

☐ Y ☐ N | Cleaned between all skin folds ______________________________

☐ Y ☐ N | Cleaned between fingers/toes ______________________________

☐ Y ☐ N | Cleaned ☐ female or ☐ male genital area well ______________________________

☐ Y ☐ N ☐ N/A | Cleaned occlusive/semi-permeable dressings ______________________________

☐ Y ☐ N ☐ N/A | Cleaned 6" of all tubes/central lines/drains closest to body ______________________________

☐ Y ☐ N ☐ N/A | Cleaned superficial wounds/rash/stage 1 & 2 decubitus ulcers ______________________________

☐ Y ☐ N ☐ N/A | Cleaned surgical wounds ______________________________

☐ Y ☐ N | Wipes/cloths replaced when soiled/as needed ______________________________

☐ Y ☐ N | Diluted bath with hot water without adding more soap ______________________________

☐ Y ☐ N | Cleaned body areas using clean-to-dirty sequence ______________________________

☐ Y ☐ N ☐ N/A | Disposed of used wipes in trash/did not flush ______________________________

☐ Y ☐ N | Resident complained about being cold during the bath ______________________________

Comments on Bathing Processes

____

____

____

____

Query to Bathing Assistant

1. **Tell me what you do during bathing when you see a wound:**

   ____

   ____

2. **Tell me how you bathe residents if they have any of the following:**

   Lines ______________________________ **Drains** ______________________________

   Tubes ______________________________ **Non-gauze dressings** ______________________________

3. **What part of your bathing style or skills are you most proud of and why?**

   ____

   ____

4. **What bathing advice would you share with a new staff member that you wish you had known earlier? How did you learn this?**

   ____

   ____

5. **How many years have you worked as a CNA in a nursing home?** ______________________
